# Implementation of telepsychiatry in Kenya: acceptability study

**DOI:** 10.1192/bjo.2022.53

**Published:** 2022-04-19

**Authors:** Loice Cushny Kaigwa, Frank Njenga, Linnet Ongeri, Anne Nguithi, Maryanne Mugane, Gathoni M. Mbugua, Jacqueline Anundo, Margaret Zawadi Kimari, Maricianah Onono

**Affiliations:** Chiromo Mental Health Hospital, Nairobi, Kenya; Chiromo Mental Health Hospital, Nairobi, Kenya; Chiromo Mental Health Hospital, Nairobi, Kenya; Chiromo Mental Health Hospital, Nairobi, Kenya; Chiromo Mental Health Hospital, Nairobi, Kenya; Chiromo Mental Health Hospital, Nairobi, Kenya; Chiromo Mental Health Hospital, Nairobi, Kenya; Chiromo Mental Health Hospital, Nairobi, Kenya; Centre for Microbiology Research, Kenya Medical Research Institute, Kisumu, Kenya

**Keywords:** Low- and middle-income countries, telepsychiatry, acceptability, patient and provider perspectives, qualitative research

## Abstract

**Background:**

COVID-19-related restrictions on in-person contact in healthcare, increasing psychiatric illness during the pandemic and pre-existing shortages of mental healthcare providers have led to the emergence of telepsychiatry as an attractive option for the delivery of care. Telepsychiatry has been promoted as economical and effective, but its acceptance in low- and middle-income countries is poorly understood.

**Aims:**

To explore the acceptance, experiences and perspectives of patients and healthcare providers in the uptake of telepsychiatry services in a middle-income country.

**Method:**

Focus group discussions were conducted on the WhatsApp platform with patients and care providers who have engaged in telepsychiatry. Data were analysed using a thematic approach.

**Results:**

Three main themes emerged from the five focus groups: (a) technical access, (b) user experience and (c) perceived effectiveness compared with face-to-face (in-person) interactions. Care providers reported challenges establishing rapport with the patient, particularly for initial sessions, maintaining privacy during sessions and detecting non-verbal cues on video. Patients cited internet connectivity problems, difficulty finding private space to have their sessions and cost as major challenges. Patients also felt in-person sessions were better for initial visits. Both patients and providers reported difficulties making insurance payment claims for telepsychiatry services. Overall, participants were mostly positive about telepsychiatry, citing its convenience and overall perceived effectiveness compared with in-person sessions.

**Conclusions:**

Telepsychiatry is an acceptable platform for delivery of out-patient psychiatric services in a middle-income country. Patients and providers appreciate the convenience it offers and would like it integrated as a routine mode of delivery of care.

COVID-19, which was declared a global pandemic in March 2020, has had several implications for mental health: interruption of care for existing patients in the context of social distancing and in some instances complete lockdown;^1^ symptom exacerbation or relapse among patients with pre-existing psychiatric illness attributable to COVID-19-related stress;^2^ and the development of psychiatric illnesses in individuals who tested positive for COVID-19 either as a result of the virus pathology (as is the case in the development of delirium, anxiety and depression)^3^ or as a result of psychological disturbances arising from the experience of illness.^4^

COVID-19 therefore presents a unique set of challenges because whereas there is an anticipated increase in the number of people requiring mental health services,^2^ the demand is coming up against an already strained mental health workforce^5^ and ardent calls for social distancing, with some areas in complete lockdown. This complex interaction of context and challenges has necessitated the adoption of digital channels in the provision and delivery of mental healthcare.^2^

Overall, in terms of outcomes, telepsychiatry has been shown to have minimal and sometimes no difference when compared with face-to-face interactions and is demonstrated as having potential for routine use.^6^

## Study justification

Although there are many studies demonstrating the effectiveness of telepsychiatry in high-income countries,^7^ few have been conducted in low- and middle-income countries;^8^ those available in the African region centred on digital interventions using phone applications (apps)^9^ and only one focused on the practice of telepsychiatry via videoconferencing.^10^ In addition, very few studies globally have examined technological feasibility, cost-effectiveness and qualitative satisfaction reports.^11^

This study therefore seeks to understand the acceptability of telepsychiatry from both a provider and patient perspective in a middle-income country – Kenya. It does so by exploring the experiences of both patients and providers with a view to understanding the experience as well as perceived effectiveness.

## Method

### Study design

This is an exploratory qualitative study that uses a grounded theory approach. Data were collected using focus group discussions. The groups were hosted virtually on the WhatsApp text platform as a measure to ensure social distancing in keeping with the government directive to limit all travel and gatherings to those deemed essential. WhatsApp focus groups have been deemed a reliable tool for effective data collection in health research.^12^

### Ethical considerations

The authors assert that all procedures contributing to this work comply with the ethical standards of the relevant national and institutional committees on human experimentation and with the Helsinki Declaration of 1975, as revised in 2008. All procedures involving human participants were approved by the Strathmore University Institutional Review Board (ref no. SU-IERC0945/20). Participants were called using an approved script where they were informed about the purpose of the study. It was made clear that their participation would be voluntary and they would be free to exit the focus group at any point. They were also informed that there would be a small risk of identification owing to visibility of their phone numbers. Other identifiable information, such as pictures and names, would be removed. To mitigate the risk of identification, we asked that participants abide by an honour code not to contact other members of the focus group at a later date or take screenshots. After verbal consent was witnessed and formally recorded, participants were enrolled. They were next contacted right before they were sent the link to the WhatsApp focus group to re-confirm consent and identity.

### Study site

The study was conducted at a private mental health facility located in Nairobi offering both in-patient and out-patient services. The facility has since expanded to five branches but at the time of the study it had four branches with a combined in-patient capacity of 200 and seeing 200–300 patients in the out-patient facility daily. It is the largest private mental health facility in East and Central Africa, so findings from this study can inform private care.

### Study population and sample size

Potential participants had to meet the following inclusion criteria:

- be able to express themselves clearly;
- have access to a smartphone with WhatsApp installed;
- be between 18 and 50 years of age; and
- have had an experience of both in-person and remote therapeutic sessions.

The study used five WhatsApp focus groups. Four of these groups (patient groups 1–4) comprised patients and the fifth group (Chiromo Lane Medical Centre (CLMC) group) comprised healthcare providers – both psychologists and psychiatrists. The four patient groups had an emphasis on homogeneity of gender and age within the groups and heterogeneity between the groups to increase variability and depth. The fifth group, made up of the healthcare providers, was heterogeneous in terms of gender and age. The groups are as shown in Table 1.

**Table 1:** Distribution of study participants

Study group	Gender	Age, years
Group 1 (patients)	Female	18–34
Group 2 (patients)	Female	35–50
Group 3 (patients)	Male	18–34
Group 4 (patients)	Male	35–50
Group 5 (healthcare providers)	6 female and 1 male	28–72

The discussions were facilitated by a moderator (L.C.K.), who is a qualified research fellow trained in research methods, and psychologists trained in effective moderation and familiar with the patients; the facilitators followed a discussion guide.

Sampling for the patient groups was purposive to include males and females between 18 and 50 years of age as well as healthcare providers who had provided mental health services digitally. Identification of the study participants was through collaboration and guidance by the management of the facility.

### Study variables

The discussion explored internet connectivity, cost of connection, quality of connection, familiarity with digital tools, factors influencing the choice of device, ability to use the chosen device, level of comfort with use of telepsychiatry (including concerns regarding privacy) and perceived effectiveness compared with face-to-face interactions.

### Study procedures

After purposive sampling, 40 participants were identified, to allow for five groups of about eight each. The selected participants were called using the script approved by the ethics board and asked for their verbal consent to participate in the study. The participants were then asked to abide by a code of honour not to divulge any of the information shared in the focus groups. Once the potential participant accepted, we informed them of the day and time that they were required to participate. At the designated time of the focus group discussion, participants were called again to confirm identity and confirm consent. The link to join the WhatsApp group was then sent to each participant's phone number and clicking on the link to join the group was considered explicit consent to participate in the study. This was clearly outlined in the message preceding the link.

The date and time of participation was predetermined, and the participants were added to the group at the set time. Participants were also asked to remove their WhatsApp profile pictures and names to minimise the amount of identifying information. The code of honour was then reiterated and then the discussion would begin. Facilitators mostly kept within the allotted time (1 h) using a discussion guide to steer the discourse. Once the time was up, the facilitator ended the discussion and thanked the participants. The facilitator then removed all participants from the group, exported the chat to the data analyst, and then deleted the group from his or her device.

Throughout the study processes (recruitment and discussions), we practised reflexivity by continually examining our own biases, preferences and theoretical predisposition and how those could play a role in our understanding and interpretation of what we were evaluating.

### Data management and analysis

Data analysis was done continuously during the initial data collection period by researchers well trained in medical research and social sciences. Data saturation was also discussed and it was concluded that data saturation was reached. We (all the authors) first familiarised ourselves with the data by reading the transcripts and noting initial ideas. Once familiar with the data, L.C.K. and L.O. identified preliminary codes from data that appeared meaningful and interesting. We acknowledge that the codes identified were influenced by background literature and the researchers’ experiences and values. We used comparing and contrasting techniques^12^ to identify and define codes, assign data to different codes and search for atypical data that did not fit a particular code. Theoretical sampling was also used to focus and generate data to feed the iterative process of continual comparative analysis of the data.^13^ This process led to the identification of broad themes from the data. Transcripts were then fine coded a second time using Dedoose (Sociocultural Research Consultants, Los Angeles, California), an online qualitative software program for analysing qualitative data through masked (‘blinded’) double coding.^14^ The platform was run on Windows. Discrepancies between the two coders (L.O. and L.C.K.) were discussed with other researchers for feedback until consensus was established. Relevant data excerpts were extracted according to the previously defined themes and typical statements were used for citation.

## Results

A total of 37 participants were interviewed in five focus groups. Each focus group had between 6 and 8 participants. Three main themes emerged from the five groups: technical access, user experience and perceived effectiveness in meeting therapeutic goals.

### Technical access

#### Availability of internet and type of connection (data bundles, Wi-Fi or cable (office connection))

For connectivity purposes, most respondents from the patient population used Wi-Fi, particularly when they were in their homes. They reported using regular calls and mobile data when outside their homes:

‘I use WIFI and sometimes normal calls’ (patient group 3, respondent R6)

‘At the clinic Wi-Fi. Bundles at home’ (patient group 2, R4).

Healthcare providers cited ethernet and office Wi-Fi as their main source of internet access for therapeutic calls:

‘All the above including even land-line phone’ (CLMC group, R3).

#### Quality of connection (picture and sound quality)

Although there were no connectivity problems for the majority, both patients and providers reported that from time to time they would experience some connectivity problems:

‘For me the picture and audio were good, but sometimes it could hang either from my side or from the doctor/therapist side’ (patient group 3, R2)

‘It depends on what the client is using and the connectivity. Sometimes the client does not know how to position the camera. All the same, it occasionally allows for a light moment and we laugh and it helps the client to relax’ (CLMC group, R4).

Both clients and therapists sought alternative communication and connectivity measures in some instances where WhatsApp, Zoom and other videoconferencing platforms were unreliable and frustrating. In these instances, phone calls were considered more effective:

‘The first time we used it, it kept hanging. Then we hang up to connect again – I call, the counsellor calls, till we just gave up. Now we don't bother, we just call directly’ (patient group 1, R4)

‘I mostly used more reliable phone calls. Zoom or videoconferencing works but the frustration from inconsistent connections is a lot’ (patient group 4, R2)

‘There were connectivity issues via Zoom and it took a while to set up the call and I prefer phone calls’ (patient group 4, R1).

#### Choice of device

Phones were the most commonly used device for the telepsychiatry sessions. Even though some respondents were using their laptops and computers, especially when they were in their offices, it was not as often as phone usage:

‘My phone always’ (patient group 3, R7)

‘Phone primarily, laptop a few times’ (patient group 2, R3)

‘Phone and personal computer’ (patient group 1, R1)

‘Phone and laptop mostly but when in office the desktop’ (CLMC group, R2).

#### Platforms used

WhatsApp, Zoom and Google Meet were the platforms most commonly used for the video therapy sessions. Regular phone calls and WhatsApp calls were also widely used in scenarios where connectivity was problematic:

‘WhatsApp, then migrated to regular calls’ (CLMC group, R2)

‘Duo video call or WhatsApp video call’ (patient group 2, R5)

‘Zoom mostly, Google Meet once’ (patient group 4, R1)

‘We used Duo and normal phone calls’ (patient group 1, R3).

### User experience

#### Level of comfort with audio-video interaction

Many of the respondents from the patient groups were comfortable with online contact, with most preferring video sessions over audio sessions when there were no limitations in connectivity. They perceived it as facilitating a better therapeutic connection with the healthcare provider:

‘I was OK – loved it and was comfortable’ (patient group 3, R3)

‘I am comfortable connecting online’ (CLMC group, R6)

‘It was comfortable ’cos you feel connected and at ease’ (patient group 1, R2).

Respondents from the healthcare provider group also indicated that, after some initial discomfort, they now felt comfortable conducting online sessions:

‘Initially I had a bit of discomfort, especially during webinars when all participants had their videos and mics muted, but now am very comfortable with it’ (CLMC group, R1).

#### Perceptions of confidentiality and privacy

Most patients said that they felt that the sessions were confidential (the provider was in an environment where the patient did not feel like there were third parties listening or watching):

‘Yes. I felt that the session was confidential’ (patient group 1, R4)

‘Yes, it was, it is confidential and you feel free to talk to the counsellor on all issues in your mind’ (patient group 1, R3).

Finding a quiet and private space while at home or work was recognised by some patients as a barrier for the sessions. Providers also indicated that patients sometimes experienced frequent interruptions and distractions:

‘This was a challenge. Being home and having to find a place with no distractions’ (patient group 4, R2)

‘Like once, a man walked in my client's session and totally disrupted the flow because she became very guarded yet we were making good progress. She has never told me who that guy was’ (CLMC group, R3).

#### Cost

According to the focus group discussion with the healthcare providers, the cost of therapeutic sessions carried out online is the same as that of an in-person session:

‘Yes, we charge a call session the same as we would charge a full session’ (CLMC group, R6).

For patients on healthcare insurance this presented a challenge and sometimes a complete barrier as the insurance covers they were using did not cover online therapy:

‘Perfect point – I have that challenge as well. My sessions would be not that spaced if insurance could pay. Sometimes I need a session but, well, the pocket can't stretch so I skip it’ (patient group 4, R6).

For some, however, the online sessions were more affordable than the typical therapy set-ups as they did not have the added cost of commuting:

‘I mostly use WhatsApp video call but in case of not having Wi-Fi I switch to a normal voice call. I chose them because of convenience and affordability’ (patient group 3, R6).

Overall, for short consultations, there were no provider-associated costs for either self-paid or insured patients:

‘Not really. Unless it is agreed that it is a session. Many times patients call briefly for quick advice […] no charge unless a prescription is issued. Prescription alone has less charge’ (CLMC group, R3)

‘On my end too because after a call I then request for a follow-up session either physically or through e-sessions’ (CLMC group, R6).

#### Convenience

The majority of respondents from the patient groups said that telepsychiatry was more convenient than in-person (‘physical’) sessions since they could attend the sessions in the comfort of their home or workplace. This eliminated the expense of time used in travel and travel costs. Some respondents cited travel time, distance and cost as the major impediments for seeking help in managing some symptoms of addiction, thus making telepsychiatry an attractive alternative:

‘The biggest thing for me is the convenience of not having to go all the way to a physical location. We live quite far so it was a relief not to have to come to the facility. Also in the state I was in, I would have my sessions in my pajamas’ (patient group 3, R1)

‘I love that I can just call and get my sessions done. I work across town and […] physical sessions were hard to plan to attend’ (patient group 1, R3)

‘Especially for drug reviews – can't be beaten for convenience. A lot of people get locked out when travel time and expense are a barrier to support. Especially when your symptoms make seeking help so much harder’ (patient group 1, R2).

### Perceived effectiveness in meeting therapeutic goals

Most respondents from the patient groups felt that the goals and objectives for each therapeutic session were met. For those who felt their needs were not fully met, disruption in internet connectivity was cited as the most common reason:

‘Yes, my needs were met’ (patient group 2, R1)

‘Yes, they were met’ (patient group 4, R2)

‘Most definitely’ (patient group 3, R3).

One participant remarked that, owing to the nature of their illness, they found it useful to record critical issues:

‘I was able to express all the things that I needed to. However, session goals were hard to achieve because I was depressed in that season so I would be given work to do and I would not be able to do it […]. Sometimes yes, other times you remember some things you could have asked that skipped the attention. With time, I learned to write the critical issues down for better follow-up’ (patient group 1, R1).

Some respondents felt that in-person sessions were more effective than remote sessions as the therapist could more easily pick up patients’ non-verbal cues, which could be essential for the session. Respondents (both patients and providers) felt that it was best to have initial sessions in person, especially for patients who were starting therapy, to establish rapport and then have follow-up sessions remotely:

‘To an extent, yes. However, I found it easier to open up and discuss more in personal one-on-ones. In the call sessions, I felt I at times brushed through a few discussion topics without going deeper into the matter’ (patient group 4, R3).

The first consultation was found to be more effective when conducted in an in-person session. This also allowed the clinician to pick up on non-verbal communication that may be curtailed or minimally obtained in a telepsychiatry session:

‘I prefer face-to-face, especially with new clients. For follow-up I feel both can work equally well’ ‘CLMC group, R2)

‘It is useful when distance is a hindrance but face-to-face in-person settings seem more effective to me in establishing rapport and maintaining a provider–patient relationship’ (CLMC group, R6).

Some respondents felt that in-person sessions were more private than the telepsychiatry sessions, especially when they were conducted in in-patient facilities:

‘Whenever I meet with the doc (not a psychologist) they set up in a consultation room and not in my room. I therefore use their computer where a nurse is always present. I prefer one-on-one in my privacy’ (patient group 3, R5).

## Discussion

The first theme identified – technical access, which includes access to internet services and internet-enabled devices – confirms findings in previous literature that access to technology and reliable connectivity influence the uptake of telehealth services and that this is especially a concern in low- and middle-income countries.^15^ The results from this study show that the widescale adoption of mobile technology among the Kenyan population,^16^ as well as the increased access to reliable internet, make telepsychiatry a viable healthcare delivery model.

The second theme – user experience – was marked by the following parameters: whether both patients and providers were comfortable with screen contact, perceptions of privacy and security, cost (affordability) and convenience. Most patients expressed comfort with screen contact and felt that the sessions were confidential in the sense that the information they shared with the provider was kept confidential. This is consistent with previous findings.^17^ Privacy, however, was a major concern, particularly as patients did not always have access to secure spaces where they could share their concerns freely. This has also been demonstrated in previous research.^18^ Respondents in this study did not raise any concerns regarding security, i.e. access to the sessions by third parties or hacking. Previous studies have shown that security has been a concern in other settings.^19^

Most patients perceived the cost of telepsychiatry to be lower, which is consistent with published findings that note the direct and indirect costs to be lower than those of face-to-face interactions.^7^ However, some patients who rely on healthcare insurance sometimes had to pay out of pocket. Providers also had problems following up on payments for online services rendered. This has also been reported in other settings and there is a need to establish a clear framework governing compensation and claims for telepsychiatry services.^20^ A majority of patients also indicated that the telepsychiatry services were more convenient than face-to-face interactions as there was no need to commute and the sessions were more likely to be on time than in-person sessions, which did not always begin according to schedule. This reflects the results of other studies examining convenience.^21^

In terms of the third theme – perceived effectiveness – respondents felt that their therapeutic needs were met. This is consistent with various studies done over recent decades in which the effectiveness of telepsychiatry has been examined. With respect to treatment outcomes, empirical evidence suggests that telepsychiatry is on par with face-to-face treatment for various psychiatric disorders, including attention-deficit hyperactivity disorder (ADHD), anxiety disorder, depression and post-traumatic stress disorder.^22–24^ Some healthcare providers expressed concern that the lack of in-person interaction in telepsychiatry could hinder the development of a healthy therapeutic alliance, which is often conceptualised as an emotional bond between patient and therapist and as collaboration and consensus between the two parties on the goals and tasks of the therapy.^25^ This sentiment has been echoed in a previous study,^22^ with the proposed solution by healthcare providers in this study to have initial sessions as face-to-face interactions and then subsequent sessions virtually being similarly echoed.

### Limitations

The study was conducted in a private mental health facility where patients may have more resources and are therefore able to access both the devices to participate in telepsychiatry sessions and the disposable income that allows them to purchase airtime and data bundles. The findings may therefore not be generalisable to settings lacking these means (devices and airtime). The findings can, however, guide application in private mental health facilities. Private providers are increasingly offering mental health services in Kenya, particularly for substance use disorders.^26^

Social desirability has been identified as one of the limitations of focus group discussions and as such constitutes a limitation of this study. On the other hand, online focus group discussions have been demonstrated to be feasible for collecting qualitative data in hard-to-include populations, and evaluations seem to indicate that the online group discussions give participants an opportunity to articulate their experiences and views in a way they might not have done in a traditional face-to-face group discussion.^27^

The study was conducted at a time when the lockdown measures as well as the directive for social distancing were in full effect. Consequently, the take up of telepsychiatry was high because the options for in-person sessions were limited. Future studies could look at the acceptability of telepsychiatry outside the context of stringent social distancing measures.

### Recommendations

Telepsychiatry was noted to be an effective method of providing mental healthcare. It is convenient and enables patients to seek care with minimal stigma. The results also showed that there are several practical factors that should be considered to optimise telepsychiatry sessions. These are as follows.

#### Before the session

The healthcare provider should ensure that patients’ appointments are well scheduled to avoid any overlap in session timings. The provider can use various digital tools (such as Google Calendar) to schedule sessions and give timely reminders. There are also exclusive telemedicine platforms that afford both scheduling and audio-visual communication via a central app.

The provider and patient should also ensure that their internet connection is stable and their preferred platforms are fully functional prior to the session, to minimise interruptions.

It would also be useful for patients to be aware of the therapeutic goals before the session to ensure that they are met.

#### During the session

The provider and patient should make every effort to ensure that there is privacy in the physical spaces in which they are taking the call. This means preparing to have the room beforehand but also restricting movement in and out of the space. They should ensure that their cameras are well positioned and the microphones are functioning properly to allow for the provider to pick on non-verbal cues and minimise interruptions.

Both the patient and the care provider should refer to the therapeutic goals to guide the discussion.

#### After the session

Both providers and patients lamented the lack of recognition of telepsychiatry sessions by insurance companies. Often, patients had to pay for telepsychiatry sessions out of pocket or make the commute to the facility to fill out paperwork – this defeats the purpose of convenience and makes the services unattainable for some owing to cost restrictions. The recommendation is for the recognition of telepsychiatry sessions as effective therapeutic sessions and the adoption of remote claims systems rather than in-person or paper-based systems.

## Data Availability

The data that support the findings of this study are available from the corresponding author on reasonable request.
